# Supplementary material for: Complexome profiling of the Chlamydomonas psb28 mutant reveals TEF5 as an early PSII assembly factor
Source: Plant Cell. 2025 Mar 17;37(6):koaf055. doi: 10.1093/plcell/koaf055 (PMC12164590; doi:10.1093/plcell/koaf055)
Supplement: koaf055_Supplementary_Data [file koaf055_supplementary_data.zip › psb28_interactive.zip!/plots/intensity/qualityPlots/peptides/MDN1_Cre03.g194850.t1.2.html]

### Cre03.g194850.t1.2

Trivial name: MDN1  
SeqCoverage [median]: 45.10  
Mass: 36.60 kDa  
  
 

| Sequence | MissedCleavage | Mass [Da] | wtIntensity | muIntensity | Score |
| --- | --- | --- | --- | --- | --- |
| AGKGSATLSMAYAAAR | 1 | 1524.77 | 1083.96 | 2150.07 | 128.06 |
| ALVEAVAK | 0 | 799.48 | 7086.72 | 18202.26 | 130.77 |
| AMTDKIANAGTVVVEAK | 1 | 1716.91 | 1100.75 | 1316.13 | 67.64 |
| ANTFVSEAK | 0 | 965.48 | 21996.19 | 25284.61 | 135.43 |
| DDLFNTNAGIVK | 0 | 1305.66 | 8224.11 | 28278.93 | 147.74 |
| GADLIVIPAGVPR | 0 | 1276.75 | 2016.59 | 0.00 | 84.21 |
| GSATLSMAYAAAR | 0 | 1268.62 | 2657.35 | 7514.31 | 174.98 |
| IANAGTVVVEAK | 0 | 1170.66 | 5079.05 | 21670.60 | 192.14 |
| KPGMTRDDLFNTNAGIVK | 2 | 1976.01 | 0.00 | 677.33 | 65.72 |
| KVIGVTSLDIVR | 1 | 1298.79 | 3014.69 | 20291.83 | 101.60 |
| LAGVYDPK | 0 | 861.46 | 4612.90 | 14885.12 | 94.41 |
| VIGVTSLDIVR | 0 | 1170.70 | 8215.20 | 15365.04 | 121.73 |
| VLLGPNGVAK | 0 | 966.59 | 778.90 | 3263.69 | 81.64 |
| VMGLGELDAFEQAAMAAMLPQLK | 0 | 2433.21 | 1498.94 | 0.00 | 140.20 |
|  |  |  | 67365.3 | 158899.9 |  |
